# Cerebral Autoregulation Indices Are Not Interchangeable in Patients With Sepsis

**DOI:** 10.3389/fneur.2022.760293

**Published:** 2022-03-07

**Authors:** Juliana Caldas, Armin Alvaro Quispe-Cornejo, Ilaria Alice Crippa, Carles Subira, Jacques Creteur, Ronney Panerai, Fabio Silvio Taccone

**Affiliations:** ^1^Escola Bahiana de Medicina e Saúde Pública, Salvador, Brazil; ^2^Universidade de Salvador, Universidade y Faculdade Salvador (UNIFACS), Salvador, Brazil; ^3^Instituto D'Or de Pesquisa e Ensino (IDOR), Salvador, Brazil; ^4^Department of Intensive Care Unit, Erasme Hospital, Université Libre de Bruxelles, Brussels, Belgium; ^5^Department of Intensive Care Medicine, Alhaia Xarxa Assistencial Universitaria de Manresa, Barcelona, Spain; ^6^Department of Cardiovascular Sciences, University of Leicester, Leicester, United Kingdom; ^7^National Institute for Health Research (NIHR) Leicester Biomedical Research Centre, Leicester, United Kingdom

**Keywords:** correlation, autoregulation index, Mxa, cerebral autoregulation, sepsis

## Abstract

**Introduction:**

Dynamic cerebral autoregulation (dCA) is frequently altered in patients with sepsis and may be associated with sepsis-associated brain dysfunction. However, the optimal index to quantify dCA in patients with sepsis is currently unknown.

**Objective:**

To assess the agreement between two validated dCA indices in patients with sepsis.

**Methods:**

Retrospective analysis of prospectively collected data in patients with sepsis; those with acute or chronic intracranial disease, arrhythmias, mechanical cardiac support, or history of supra-aortic vascular disease were excluded. Transcranial Doppler was performed on the right or left middle cerebral artery (MCA) with a 2-MHz probe, and MCA blood flow velocity (FV) and arterial pressure (BP) signals were simultaneously recorded. We calculated two indices of dCA: the mean flow index (Mxa), which is the Pearson correlation coefficient between BP and FV (MATLAB, MathWorks), and the autoregulation index (ARI), which is the transfer function analysis of spontaneous fluctuations in BP and FV (custom-written FORTRAN code). Impaired dCA was defined as Mxa >0.3 or ARI ≤ 4. The agreement between the two indices was assessed by Cohen's kappa coefficient.

**Results:**

We included 95 patients (age 64 ± 13 years old; male 74%); ARI was 4.38 [2.83–6.04] and Mxa was 0.32 [0.14–0.59], respectively. There was no correlation between ARI and Mxa (*r* = −0.08; *p* = 0.39). dCA was altered in 40 (42%) patients according to ARI and in 50 (53%) patients according to Mxa. ARI and Mxa were concordant in classifying 23 (24%) patients as having impaired dCA and 28 (29%) patients as having intact dCA. Cohen's kappa coefficient was 0.08, suggesting poor agreement. ARI was altered more frequently in patients on mechanical ventilation than others (27/52, 52% vs. 13/43, 30%, *p* = 0.04), whereas Mxa did not differ between those two groups. On the contrary, Mxa was altered more frequently in patients receiving sedatives than others (23/34, 68% vs. 27/61, 44%, *p* = 0.03), whereas ARI did not differ between these two groups.

**Conclusions:**

Agreement between ARI and Mxa in assessing dCA in patients with sepsis was poor. The identification of specific factors influencing the dCA analysis might lead to a better selection of the adequate cerebral autoregulation (CAR) index in critically ill patients with sepsis.

## Introduction

Sepsis is a common cause of admission to the intensive care unit (ICU), with a great impact on mortality (1). Sepsis-associated brain dysfunction (SABD), ranging from delirium to coma, is common during sepsis and can be associated to poor outcomes (2). The pathophysiology of encephalopathy occurring during sepsis remains unclear, but likely involves alterations in neurotransmission, microglial activation, and blood–brain barrier dysfunction (3). Cerebral hypoperfusion may also play a role, indeed, cerebral blood flow (CBF) may be inadequate secondary to microcirculatory dysfunction (4, 5). Cerebral autoregulation (CAR) is the intrinsic cerebrovascular mechanism that maintains CBF constant within different ranges of cerebral perfusion pressure (CPP); indeed, cerebral arterioles can constrict or dilate in response to the elevation or reduction in CPP, the so-called pressure autoregulation, thus keeping CBF within stable values. As CPP is determined by the interaction between intracranial pressure and mean arterial pressure (MAP), the latter can be used as a valid surrogate of CPP in those patients in whom intracranial pressure is not expected to be elevated (6). Mechanisms of autoregulation are efficient for a range of MAP, which varies between subject, but is considered to be around 50–150 mmHg; as a consequence, alterations in CAR may result in brain hypoperfusion at MAP levels, which are considered to be adequate in routine practice (5). During sepsis, an impaired CAR has been reported in several studies (5, 7–10); such disturbances have been associated with increased serum concentrations of brain injury biomarkers (11) and with the occurrence of brain dysfunction (5).

Several studies investigating alterations of CAR in patients with sepsis and non-sepsis have been published. However, they differed in timing of autoregulation assessment, technique of assessment, and alteration measurements and definition. The autoregulation index (ARI) (12) and the mean flow index (Mxa) (13) have been validated in patients with critical illness. Both are based on spontaneous fluctuation in MAP and its correlation with intracranial artery blood flow velocity (FV) as measured by transcranial Doppler (TCD) (14). As no specific index is currently considered to be the “gold standard” in patients with critical illness, the classification of CAR based on different indices may result in divergent results when applied to the same study cohort. However, no study has previously compared ARI and Mxa in patients with sepsis.

The aim of this study was to compare the assessment of CAR using ARI and Mxa in a large cohort of patients with sepsis.

## Methods

### Study Population and Data Collection

Retrospective analysis of prospectively collected data including adult (>18 years) patients who were diagnosed with sepsis either on admission or during ICU stay at Erasme University Hospital (from October 2018 to December 2020; Université Libre de Bruxelles, Belgium) and Althaia Foundation Hospital (from June 2012 to June 2015; University of Manresa, Barcelona, Spain), and who had a TCD performed within 72 h from diagnosis (Table 1). Exclusion criteria were chronic or acute cerebrovascular disease (i.e., ischemic or hemorrhagic stroke), history of supra-aortic vascular stenosis, cardiac arrhythmias, mechanical cardiac support (i.e., veno-arterial extracorporeal membrane oxygenation, left ventricular assist device, intra-aortic balloon pump counter-pulsation), severe hypotension (MAP <50 mmHg), severe hypercapnia (i.e., PaCO_2_ > 65 mmHg), pregnancy, moribund patient or withdrawal of life-sustaining therapy, absence of transtemporal bone window for TCD examination, and absence of invasive arterial BP monitoring. We collected demographic data, Acute Physiology and Chronic Health Evaluation II (APACHE II) score, site of infection, the pathogen(s) involved, and the outcome at ICU discharge. Use of sedation and/or neuromuscular blocking agents (NMBAs), vasopressors, and mechanical ventilation at the moment of CAR assessment was also recorded. The study protocol was approved by local ethics committees; due to the retrospective and anonymous nature of the study, the need for an informed consent was waived.

**Table 1:** Characteristics of the study population (*n* = 95). Data are presented as count (%), mean (±SD), or median [25th−75th percentiles].

Age, years	64 (13)
Male gender, *n* (%)	70 (74)
APACHE II score on admission	23 [15–32]
ICU length of stay, days	6 [3–14]
ICU mortality, *n* (%)	75 (79)
Car indices
ARI	4.38 [2.83–6.04]
Mxa	0.32 [0.14–0.59]
At time of car assessment
Sedation, *n* (%)	34 (36)
NMBA, *n* (%)	13 (14)
Mechanical ventilation, *n* (%)	52 (55)
Vasopressors, *n* (%)	69 (73)
Infection source
Abdominal, *n* (%)	42 (44)
Respiratory, *n* (%)	26 (27)
Urinary tract, *n* (%)	7 (7)
Others, *n* (%)	20 (21)
Pathogen
Bacterial, *n* (%)	66 (70)
Fungus, *n* (%)	9 (10)
Virus, *n* (%)	3 (3)
Unknown, *n* (%)	17 (18)

*APACHE II, Acute Physiology Age Chronic Health Evaluation II; ICU, intensive care unit; LOS, length of stay; Mxa, mean flow index; dCA, dynamic cerebral autoregulation; NMBA, neuromuscular blocking agent; CAR, cerebral autoregulation*.

### Cerebral Autoregulation Assessment

Transcranial Doppler was performed on the right or the left middle cerebral artery (MCA) using a 2-MHz, 100 Hz sampling TCD monitoring probe (Compumedics DWL, Germany), which was kept in place using a special helmet to ensure a constant angle of insonation for 8 min length recording at the bedside. FV and invasive arterial BP signals were downloaded on a personal computer. Patients were maintained in steady-state conditions throughout the examination. Modifications in respiratory settings and/or pharmacological or fluidic therapy were avoided either before or during TCD examination. Samples were automatically (by a custom-written script) and visually expected for artifacts (e.g., due to tracheal suctioning, arterial line flushing, or transducer malfunction); in case of artifacts, the entire cardiac cycle was discarded; in case of artifacts >10% of the total recording, the entire recording was discarded.

The Mxa is the Pearson correlation coefficient between BP and FV and was calculated as previously reported (5, 13), i.e., both signals were averaged on 10-s consecutive windows with 50% overlap for the entire length of the recording; therefore, the correlation coefficient was calculated using MATLAB (MathWorks, Natick, MA, USA). The Pearson correlation coefficient (*r*) represents the strength and direction of a relationship between two variables. It has a value between +1 and −1, where +1 represents a total positive correlation, 0 represents no correlation, and −1 represents total negative correlation. The correlation is considered to be moderately positive when *r* > 0.3. Given that changes in FV mirrors changes in CBF, Mxa > 0.3 means that CBF is dependent on BP changes and dynamic cerebral autoregulation (dCA) is impaired; when BP and FV have a weak or a negative correlation (Mxa ≤ 0.3), dCA is considered intact.

Autoregulation index was calculated as follows: all signals were low-pass filtered using an eighth-order Butterworth with a cutoff frequency of 20 Hz. Beat-to-beat parameters were interpolated with a third-order polynomial and resampled at 5 Hz to generate signals with a uniform time base. The Welch method was adopted for smoothing spectral estimates obtained with the fast Fourier transform (102.4 s segments, 50% superposition) (15). An interpolation procedure was adopted to obtain ARI values (ARI = 0 indicates absent dCA, whereas ARI = 9 corresponds to the most efficient dCA) that can be observed by fitting a second-order polynomial to the integer values of ARI neighboring the region of minimum error (15). Objective criteria were adopted for the acceptance of estimates of ARI, using the normalized mean square error for fitting the Tiecks model (15) to the FV step response and the 95% CI for the mean coherence function in the frequency interval 0.15–0.25 Hz (16). ARI ≤ 4 or Mxa <0.3 defined “impaired” dCA (14, 16).

### Statistical Analysis

Data were tested for normality using the Kolmogorov–Smirnov test. Categorical variables were compared using the Fisher exact test or chi-square test, as appropriate, and the Student *t*-test or the Mann–Whitney *U*-test was used to compare continuous variables, as appropriate. The correlation between ARI and Mxa was assessed using Pearson's coefficient. Cohen's kappa (κ) coefficient (17) defined agreement between ARI and Mxa classification of dCA as either altered or intact: agreement was defined poor if κ <0.2; fair if 0.21 < κ <0.4; moderate if 0.41 < κ <0.6; substantial if 0.61 < κ <0.8; almost perfect if κ > 0.8. If ARI and Mxa were concordant, patients were categorized as having “impaired” dCA or “intact” dCA, accordingly; a third group, named “divergent” dCA included those patients for whom the classification was not concordant between the two indices. dCA according to ARI and Mxa was assessed in different subgroups of patients according to: (a) mechanical ventilation; (b) administration of sedatives; (c) administration of NMBAs; (d) use of vasopressors at the time of TCD assessment; and (e) ICU outcome. Statistical analysis was performed using SPSS (IBM SPSS Statistics 25.0 for Macintosh). For all statistical tests, the significance was set at *p* < 0.05. Data are presented as median (25th−75th percentiles) or mean ± SD, as appropriate. Categorical variables are presented as counts (%).

## Results

A total of 95 patients (mean age 64 years old; male 74%) were eligible over the study period; APACHE II score on admission was 23 [15–32], and the ICU length of stay was 6 [3–14] days. Most infections were due to bacteria (*n* = 66, 70%), and mostly affected the abdomen (*n* = 42, 44%) and the lungs (*n* = 26, 28%). ICU mortality was observed in 20 (21%) patients.

**Figure 1:**
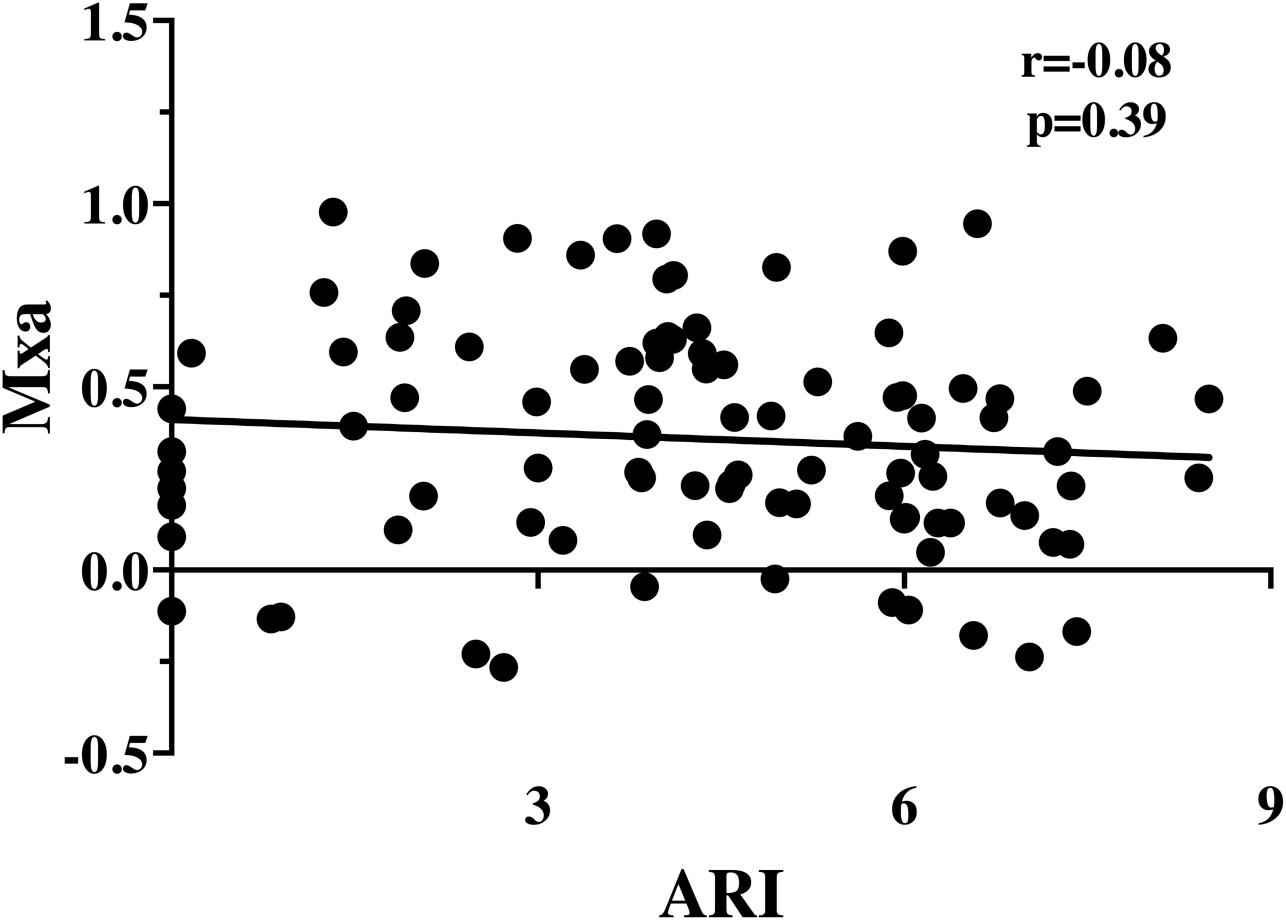
Correlation between autoregulation index (ARI) and mean flow index (Mxa).

At the dCA assessment, sedatives, neuromuscular blocking agents, mechanical ventilation, and vasopressors were used in 34 (36%), 13 (14%), 52 (55%), and 69 (73%) patients, respectively. Median ARI and Mxa values were 4.38 [2.83–6.04] and 0.32 [0.14–0.59], respectively; there was no significant correlation between ARI and Mxa (*r* = −0.08; *p* = 0.39; Figure 1). Impaired dCA according to the ARI threshold was observed in 40 (42%) patients; impaired dCA according to Mxa threshold was observed in 50 (53%) patients. In particular, ARI and Mxa were concordant in classifying 23 (24%) patients with impaired dCA and 28 (29%) patients with intact dCA (Table 2); a poor agreement between the two indices to categorize dCA was therefore obtained (Cohen's kappa coefficient = 0.08).

**Table 2:** Comparison of altered and intact cerebral autoregulation using autoregulation index (ARI) and mean flow index (Mxa) thresholds.

		**Mxa**		
		**Altered**	**Preserved**	**Total**
ARI	Altered	23	17	40
	Preserved	27	28	55
	Total	50	45	95

There were no differences in clinical variables according to different ARI and Mxa combinations (Table 3). ARI was altered more frequently in patients on mechanical ventilation than others (27/52, 52% vs. 13/43, 30%, *p* = 0.04), whereas Mxa did not differ between these two groups. On the contrary, Mxa was altered more frequently in patients receiving sedatives than others (23/34, 68% vs. 27/61, 44%, *p* = 0.03), whereas ARI did not differ between these two groups. No other differences were found between subgroups (Table 4).

**Table 3:** Differences between patients according to the combination of mean flow index (Mxa) and autoregulation index (ARI) values (see “Methods” Section).

	**All patients**	**Altered CAR**	**Divergent CAR**	**Intact CAR**	***p*** **-value**
	**(***n*** = 95)**	**(***n*** = 23)**	**(***n*** = 44)**	**(***n*** = 28)**	
Age, years	64 (13)	65 (14)	64 (14)	63 (12)	0.86
Male gender, *n* (%)	70 (74)	17 (74)	32 (73)	21 (75)	0.99
APACHE II score on admission	23 (11)	26 (11)	23 (11)	22 (9)	0.38
ICU length of stay, days	6 [3–14]	8 [4–18]	5 [3–14]	5 [3–12]	0.29
ICU mortality, *n* (%)	20 (21)	6 (26)	12 (27)	2 (7)	0.09
At time of dCA assessment
Sedation, *n* (%)	34 (36)	11 (48)	19 (43)	4 (14)	0.14
NMBA, *n* (%)	13 (14)	3 (13)	9 (21)	1 (4)	0.11
Mechanical ventilation, *n* (%)	52 (55)	17 (74)	24 (55)	11 (39)	0.05
Vasopressors, *n* (%)	69 (73)	20 (87)	29 (66)	20 (71)	0.19
Infection source, *n* (%)
Abdominal	42 (44)	8 (35)	21 (48)	13 (46)	0.62
Respiratory	26 (27)	5 (22)	15 (34)	6 (21)	0.44
Urinary tract	7 (7)	3 (13)	1 (2)	3 (11)	0.18
Others	20 (21)	7 (30)	7 (16)	6 (21)	0.34
Pathogen, *n* (%)
Bacterial	66 (70)	14 (61)	35 (80)	17 (61)	0.14
Fungus	9 (10)	5 (22)	3 (7)	1 (4)	0.09
Virus	3 (3)	0 (0)	2 (5)	1 (4)	0.80
Unknown bug	17 (18)	4 (17)	4 (9)	9 (32)	0.29

*Data are presented as count (%), mean (SD), or median [(25^th^−75^th^) percentiles]*.

*APACHE II, Acute Physiology Age Chronic Health Evaluation II; ICU, intensive care unit; LOS, length of stay; NMBA, neuromuscular blocking agent; dCA, dynamic cerebral autoregulation; CAR, cerebral autoregulation*.

**Table 4:** Differences between impaired cerebral autoregulation according to Mxa and ARI in different subgroups of patients.

	**Altered ARI**	**Altered Mxa**	***p*** **-value**
	**(*n* = 40)**	**(*n* = 50)**	
MV *(n = 52)*	27	31	0.54
NON MV *(n = 43)*	13	19	0.26
*p*-value	0.04	0.15	
Sedatives *(n = 34)*	18	23	0.36
No-sedatives *(n = 61)*	22	27	0.42
*p*-value	0.13	0.03	
NMBA *(n = 13)*	8	7	0.99
No-NMBA *(n = 82)*	32	43	0.09
*p*-value	0.14	0.99	
Vasopressors *(n = 69)*	33	36	0.71
No vasopressors *(n = 26)*	7	14	0.12
*p*-value	0.10	0.99	
Survivors *(n = 75)*	29	37	0.22
Non-survivors *(n = 20)*	11	13	0.77
*p*-value	0.21	0.31	

*MV, mechanical ventilation; NMBA, neuromuscular blocking agent; Mxa, mean flow index; ARI, autoregulation index*.

## Discussion

In this study, we have shown that ARI and Mxa, two validated indices to assess dCA using invasive BP signal and cerebral FVs, are not inter-changeable in a large cohort of critically ill patients with sepsis. These data underline the need for further comparison between various indices of dCA in clinical practice in order to better standardize the approach to analyze this important physiological phenomenon.

Given the limited understanding of the pathophysiological processes of brain dysfunction in sepsis and the complexity of the mechanisms underlying CAR, identifying the “gold standard” index that would be suitable to quantify dCA at the bedside in these patients remains a difficult task. Over the last years, different studies have introduced new indices of dCA (i.e., based on oxygen saturation, COx; or on brain oxygen pressure, ORx), which have been “validated” not using direct measurements of CBF and their relationship to BP variations, but through the comparison with other available dCA indices (as an example, one based on intracranial pressure monitoring, PRx) (18). Although this approach has been repeatedly used in the literature, it has some important caveats: (a) a significant correlation between two indices does not imply that they have the same accuracy to define impaired dCA; (b) the presence of an acute brain injury in the studied population may affect the generalizability of one specific dCA index into a non-brain injured patients' population, as the pathophysiological mechanisms resulting in impaired dCA might differ between these two groups; and (c) the methodological characteristics to obtain some of these indices (i.e., for PRx, how long the recording should be, how many seconds the window of assessment should last, and which is the percentage of overlap between windows) are not entirely standardized and may vary across studies (19); reliability, reproducibility, and diagnostic and prognostic values are not always similar.

In our study focusing on patients with sepsis, ARI and Mxa showed a non-significant correlation, had a poor agreement to categorize the dCA impairment and even the proportion of patients identified as “impaired dCA” was different according to the used index. In the subgroup analysis, the presence of mechanical ventilation and sedation had a different impact on the determination of CAR function between the different indices. Unfortunately, we do not have a clear explanation for these findings, and some hypotheses include the use of different analytic constructs to obtain ARI and Mxa, the presence of “noise” or an inappropriate assumption, i.e., the analysis if performed at a “steady state,” whereas this is not the case in reality.

If we consider the analytic constructs underlying Mxa and ARI, both indices investigate dCA by measuring fluctuations of the MCA blood FV and arterial BP; however, Mxa is computed using a linear regression analysis, it is a non-parametric value and is not based on a predefined model (18). Moreover, Mxa describes the stability of CBF to changes in BP and quantifies how the variation of pressure would be significantly associated with variations in flow. As this approach is purely based on a time-domain measurement of dCA, Mxa has a “quasi-static” approach, since in most cases no information can be obtained about the speed of the response (20). On the contrary, ARI is based on a model in which the response of flow to a hypothetical impulse change in BP is estimated and therefore compared with the theoretical impulse response; as such, ARI explains how rapidly flow recovers after any change in pressure and is theoretically more sensitive to physiological changes than Mxa. ARI reflects both the temporal and amplitude relationship between CPP and CBF, which characterizes the dynamic dCA assessment (14, 19, 21). However, the validity of such model in different categories of patients remains unknown.

Another potential explanation is the presence of known or unknown “noise,” which can be present during recordings in different categories of patients. These factors could be minimal changes in PaCO_2_, which is a potent modulator of vascular reactivity, PaO_2_, which can also modify CBF in case of extreme oxygen values, or intracranial pressure (6). As not all patients in our cohort were on mechanical ventilation, PaCO_2_ might present significant fluctuations, therefore impacting on BF recordings. In one study, the addition of different intensity of “noise” to artificial BF and BP recordings resulted in a flattening of the relationship between ARI and Mx (22).

In one study conducted in patients with traumatic brain injury (TBI), ARI was significantly related to Mx (i.e., an index derived from CPP and FVs, which has a similar construct than Mxa), although the relationship was not linear (23). However, the authors also pointed out that both indices lost sensitivity for extreme values (i.e., close to −1 or +1 for Mx and ARI of <2). In another study from the same group focusing again on TBI, ARI, and Mx showed a significant linear relationship and correlated with outcome (18). These differences with our findings might be related to the different patients' populations (i.e., TBI vs. sepsis), the presence of brain injury and/or elevated intracranial pressure, which is extremely rare in sepsis, and different therapeutic strategies, which could result in different baseline BP values (i.e., higher in TBI than in sepsis) and lower BP variability (i.e., autonomous nervous system dysfunction during sepsis) (24) between groups. Other studies have also compared different indices among them, reporting conflicting results (18, 25–27). Only one of these studies compared different indices in patients with sepsis (28), used near-infrared spectroscopy and transfer Fourier analysis, concluding that these metrics are not interchangeable either. Future research should focus more frequently on comparison between different dCA indices in various diseases and quantify CAR through multiple analytical approaches, paying attention to their respective limitations and the caveats that must be considered.

A number of limitations of this study need to be mentioned. Our study was limited to only two of the many indices that are commonly used for the assessment of dCA. Including other indices could have identified better agreements. The application of Mxa and ARI to spontaneous BP recordings is also a limitation; it has been reported that increased variability of BP leads to more robust estimates of autoregulation (29, 30), accepting that different protocols, such as sit-to-stand or sudden release of compressed thigh-cuffs (31) can lead to different values of metrics. Nevertheless, protocols that induce significant changes in BP are not feasible in a critical care environment. Third, we routinely recorded PaCO_2_ values; PaCO_2_ is one of the strongest determinants of dCA performance (5), and it could have affected the ARI and Mxa in different ways. However, not all patients were on the controlled mechanical ventilation, and this might have resulted in variable PaCO_2_ values over the recording period for those on spontaneous breathing. Fourth, dCA was dichotomized as intact or impaired using specific thresholds proposed in previous studies; however, at least for Mxa, a threshold of 0.45 has also been suggested to better identify impaired dCA (32, 33), and healthy volunteers have also been found with Mxa values above 0.3 (33). However, the lack of correlation between absolute Mxa and ARI values would not significantly change the conclusions of our study if a different Mxa threshold would have been used to define impaired dCA. Fifth, we did not specifically assess the relationship of ARI or Mxa with the occurrence of brain complications, brain imaging, or mortality, and these analyses were beyond the scope of this investigation. Finally, Mxa could be highly dependent on the analytic approach (i.e., blocks, correlation periods, and overlaps of FV and BP), with a moderate repeatability (34); a recognized “gold standard” approach has not been identified yet.

## Conclusions

In this cohort of patients with sepsis, two of the most common indices used for the assessment of dCA, the ARI, and the Mxa, had a weak correlation and a poor agreement to classify dCA. These findings underline the limitations in comparing results on dCA from different studies, which used different analytic approaches to characterize dCA. A standardization for the dCA assessment is definitely warranted.

## Data Availability Statement

The raw data supporting the conclusions of this article will be made available by the authors, without undue reservation.

## Ethics Statement

The studies involving human participants were reviewed and approved by Comité d'Ethique Erasme-ULB. Written informed consent for participation was not required for this study in accordance with the national legislation and the institutional requirements.

## Author Contributions

AQ-C, JC, and FT conceived the study and wrote the draft of the paper. AQ-C, IC, and CS selected the population and performed the TCD. AQ-C, JC, IC, and CS collected the data. AQ-C and FT conducted the statistical analysis. FT and RP revised the text for intellectual content. The authors read and approved the final manuscript.

## Conflict of Interest

The authors declare that the research was conducted in the absence of any commercial or financial relationships that could be construed as a potential conflict of interest.

## Publisher's Note

All claims expressed in this article are solely those of the authors and do not necessarily represent those of their affiliated organizations, or those of the publisher, the editors and the reviewers. Any product that may be evaluated in this article, or claim that may be made by its manufacturer, is not guaranteed or endorsed by the publisher.
